# Sensory Modality-Dependent Interplay Between Updating and Inhibition Under Increased Working Memory Load: An ERP Study

**DOI:** 10.3390/brainsci15111178

**Published:** 2025-10-30

**Authors:** Yuxi Luo, Ao Guo, Jinglong Wu, Jiajia Yang

**Affiliations:** 1 Graduate School of Interdisciplinary Science and Engineering in Health Systems, Okayama University, 3-1-1 Tsushima-Naka, Kita-ku, Okayama 700-8530, Japan; pd257h5u@s.okayama-u.ac.jp; 2 Department of Psychology, Institute of Education, China West Normal University, Nanchong 637001, China; godcouldcry@163.com; 3 Faculty of Biomedical Engineering, Shenzhen University of Advanced Technology, No. 1, Gongchang Road, Guangming District, Shenzhen 518107, China; wujinglong@suat-sz.edu.cn

**Keywords:** working memory load, attentional resource allocation, modality-specific interference, inhibitory control, executive function, sensory modality

## Abstract

**Background/Objectives:** Working memory (WM) performance relies on the coordination of updating and inhibition functions within the central executive system. However, their interaction under varying cognitive loads, particularly across sensory modalities, remains unclear. **Methods:** This study examined how sensory modality modulates flanker interference under increasing WM loads. Twenty-two participants performed a visual n-back task at three load levels (1-, 2-, and 3-back) while ignoring visual (within-modality) or auditory (cross-modality) flankers. **Results:** Behaviorally, increased WM load (2- and 3-back) led to reduced accuracy (AC) and prolonged reaction times (RTs) in both conditions. In addition, flanker interference was observed under the 2-back condition in both the visual within-modality (VM) and audiovisual cross-modality (AVM) tasks. However, performance impairment emerged at a lower load (2-back) in the VM condition, whereas in the AVM condition, it only emerged at the highest load (3-back). Significant performance impairment in the AVM condition occurred at higher WM loads, suggesting that greater WM load is required to trigger interference. Event-related potential (ERP) results showed that N200 amplitudes increased significantly for incongruent flankers under the highest WM load (3-back) in the visual within-modality condition, reflecting greater inhibitory demands. In the cross-modality condition, enhanced N200 was not observed across all loads and even reversed at low load (1-back). Moreover, the results also showed that P300 amplitude increased with load in the within-modality condition but decreased in the cross-modality condition. **Conclusions:** These results demonstrated that the interaction between updating and inhibition is shaped by both WM load and sensory modality, further supporting a sensory modality-specific resource allocation mechanism. The cross-modality configurations may enable more efficient distribution of cognitive resources under high load, reducing interference between concurrent executive demands.

## 1. Introduction

In modern environments, individuals are frequently required to manage multiple concurrent tasks that engage different sensory inputs and cognitive systems—for example, following visual navigation instructions while processing an incoming call. Such multitasking situations impose substantial demands on cognitive control, requiring the brain to update dynamically changing task-relevant visual information while inhibiting irrelevant auditory distractions. To cope with such demands, the brain relies on a limited-capacity working memory (WM) system that supports the coordination of executive functions such as updating and inhibition to sustain goal-directed behavior, particularly in contexts requiring inhibitory control. WM serves as a central cognitive workspace: it temporarily stores, updates, and manipulates information in a way that supports flexible, goal-directed behavior. This ability to flexibly govern concurrent processing is foundational to adaptive behavior, particularly in complex or high-load environments [1]. To fully understand the neural and cognitive mechanisms that underlie such adaptive functioning, it is essential to investigate how core executive functions—specifically, updating and inhibition—interact during high-demand, parallel-task conditions. Prior studies have suggested that such interactions are modulated by both bottom-up stimulus-driven processes and top-down attentional control mechanisms [2,3,4,5,6,7,8,9,10]. In addition, WM load has been shown to either impair or facilitate cognitive control performance depending on content-specific interactions between concurrent executive functions [11,12,13,14]. These effects are further modulated by the degree of stimulus-content overlap across different WM executive function tasks [15,16,17,18]. The increased interference effect under high WM load primarily occurred when the stimuli presented in the dual task were of similar representational content (e.g., verbal–verbal). In contrast, when the representational content was dissimilar (e.g., spatial–verbal), the interference effect under a high WM load was attenuated [16]. This indicates that stimulus-specific interference effects might be explained by the existence of modality-specific attentional resources [19,20,21]. According to the influential multicomponent model of WM, visuospatial and phonological information is stored separately [22,23]. This view is supported by numerous findings of neural dissociation between phonological and visuospatial WM systems, including findings of a clear-cut double dissociation between phonological and spatial WM [24]. Moreover, each system is thought to rely on its own independent and limited attentional resources. Importantly, content-specific subsystems are believed to subserve both WM maintenance and attentional selection [16]. Therefore, many studies have suggested that when concurrent WM loads occur in the same content-specific system, the concurrent processing will compete with the within-system limited attentional resources so that each concurrent WM task with increasing load will result in impairment of the other task.

However, a critical question that has received less attention, and which this study directly addresses, is whether and how such executive interactions are modulated when tasks share the same representational content (e.g., verbal) but are delivered through different sensory modalities. From a mechanistic perspective, updating and inhibition are supported by overlapping neural networks within the frontal and parietal regions [25,26]. Their coordination is thought to depend on shared neural resources across these regions, particularly when tasks impose concurrent demands on both functions. Furthermore, sensory modality (within- vs. cross-modality) may modulate this coordination by influencing attentional allocation and representational content. Moving beyond the established paradigm of content-based interference, this study aims to specify the unique contribution of sensory modality configuration to the coordination of updating and inhibition, by examining how these interactions vary under within-modality versus cross-modality sensory conditions. In real-world environments, individuals rarely rely on a single sensory modality for information processing. Rather, healthy cognitive functioning often requires the concurrent use of multiple sensory channels to perceive and respond to complex task demands. As illustrated in the opening example of driving while navigating and talking on the phone, even simple multitasking scenarios engage multiple input systems—vision, audition, and touch. More complex situations only amplify these demands, underscoring the urgency of understanding how WM-based executive control mechanisms operate when an individual is confronted with cross-modal sensory input. Importantly, the current focus is not on multisensory integration—the merging of inputs into unified percepts—but on the ability of the brain to manage parallel streams of sensory information during concurrent executive processes. This distinction highlights the demands placed on attentional control and cognitive coordination across sensory modalities, whose inputs may remain informationally distinct yet exert reciprocal influences—either facilitative or interfering—during concurrent executive processing. This dual possibility underscores the importance of investigating how WM-based executive functions interact under within-modality versus cross-modality input conditions.

The present study builds upon and extends previous work by directly comparing the interplay between updating and inhibition in within-sensory modality (e.g., visual-visual) versus cross-sensory modality (e.g., visual-auditory) contexts. Crucially, all of the studied tasks are conducted within a verbal content domain, allowing us to isolate the effects of sensory modality from those attributable to stimulus content or representational format. This design helps clarify how sensory modality configuration modulates executive coordination under varying WM loads, thereby advancing our understanding of sensory cross-modality interference control. Extending the single-task design framework, which combines n-back and flanker tasks to minimize switching-related costs [27], the present study introduces key modifications that enable us to examine how sensory modality configurations shape the interplay between updating and inhibition under varying WM loads. Two experimental conditions were used: a within-modality condition, in which both the n-back and the flanker stimuli were presented visually, and a cross-modality condition, in which a visual n-back task was paired with auditory flanker stimuli. By comparing performance across these conditions, this study aimed to uncover the brain’s response strategies and the underlying neural mechanisms supporting interference suppression under different modality configurations.

**Figure 1:**
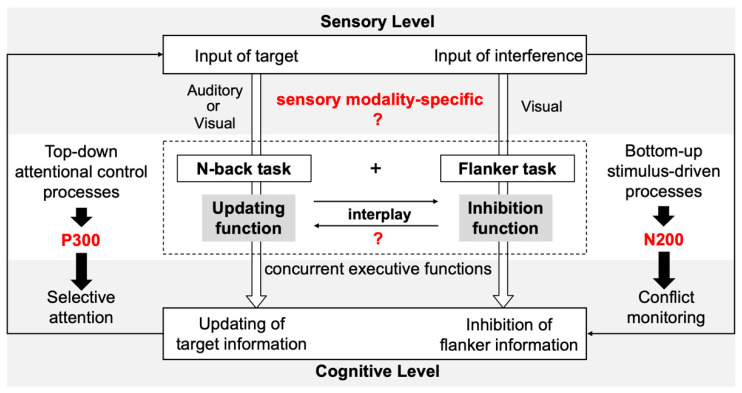
Schematic overview of the experimental design and theoretical framework. This study investigated whether the interplay between updating and inhibition functions is sensory-modality-specific by comparing visual–visual (within-modality) and audiovisual (cross-modality) conditions. The n-back and flanker tasks were combined to simultaneously engage updating and inhibition processes. At the neural level, the N200 and P300 components, respectively, indexed conflict monitoring and selective attention, reflecting how top-down control and bottom-up sensory processing jointly shape the modality-dependent coordination between executive functions.

To achieve this goal, we focused on both behavioral measures and electrophysiological markers of cognitive control and conflict processing (as shown in Figure 1). Behavioral performance was evaluated by analyzing reaction times (RTs) and accuracy (AC) rates. Prior findings demonstrated that increasing WM load (operationalized through higher n-back levels) was associated with longer RTs and lower AC, reflecting the heightened demands placed on the updating function [27,28,29,30,31,32,33]. Similarly, clear flanker interference effects have been documented, with incongruent trials eliciting significantly slower RTs and reduced AC than congruent trials do, reflecting heightened inhibitory demands [34,35,36]. Importantly, the magnitude of the congruency effect, calculated as the difference in RTs between incongruent and congruent trials, varied by type of content. Consistent with the findings for shared-resource competition, this interference effect was more pronounced in the within-content modality condition [16,18,37]. In addition to obtaining behavioral data, the present study examined two well-established ERP components that index cognitive control: N200 and P300. Both have been reliably linked to cognitive control and are known to be sensitive to WM load and flanker paradigms [38,39]. The N200 component, the largest negative-going peak occurring within the 150–300 ms latency window [40], serves as a neural index of conflict monitoring [41,42,43,44,45,46]. Prior studies have demonstrated that N200 amplitudes are enhanced during incongruent trials under high WM load conditions relative to low-load conditions, indicating intensified conflict detection when cognitive resources are taxed [39,47,48,49]. The P3 component, the largest positive-going peak occurring within the 250–500 ms latency window [50,51], reflects attentional resource allocation and context updating [40,52,53]. Compared with congruent trials, incongruent trials typically require greater cognitive control and attentional engagement, resulting in reduced P3 amplitudes—especially under high WM load conditions [27,47]. Taken together, the behavioral and electrophysiological measures obtained in this study provide complementary indices of cognitive control under varying task demands and sensory modality configurations.

On the basis of the foregoing considerations, we hypothesized that the interaction between inhibitory control and the updating of WM varies as a function of sensory modality configuration. We examined behavioral performance to test whether flanker interference effects, reflected in impairment of performance, would be more pronounced in the visual within-modality condition than in audiovisual cross-modality under varying WM loads. At the neural level, we anticipate differential modulation of the N200 and P300 components across sensory modality configurations and WM load levels, indicating the existence of sensory-specific executive coordination strategies. By systematically dissociating modality from content, our findings aim to provide a more refined framework for understanding how the brain manages concurrent cognitive demands, thereby contributing to a more complete model of working memory and cognitive control that accounts for the complexities of multisensory processing in real-world environments.

## 2. Method

### 2.1. Participants

An a priori power analysis was conducted using G*Power (version 3.1.9.7) to determine the required sample size. When the effect size was set at f = 0.25, α = 0.05, and power = 0.90 [47], the analysis recommended a minimum of 18 participants. Ultimately, 22 healthy, right-handed undergraduate or graduate students (aged 19–25 years, M = 21.36, SD = 1.56, 9 females; years of education: M = 15.36, SD = 1.22) were recruited from China West Normal University. All of the participants were native Chinese speakers, reported normal or corrected-to-normal vision and hearing, and had no history of neurological or psychiatric disorders. Written informed consent was obtained from all participants prior to the experiment. The study was approved by the Ethics Committee of the Department of Psychology, Institute of Education, China West Normal University, and was conducted in accordance with the principles set forth in the Declaration of Helsinki and institutional guidelines.

### 2.2. Stimuli

The stimuli consisted of the presentation of four capital letters (S, H, C, and F) and their corresponding pronunciations. In each trial, a centrally located target letter and two flanker letters were presented. In the visual condition, one of the four letters was randomly selected and displayed in the center of the screen. In the visual congruent condition, the same letter appeared as a flanker on both the left side and the right side of the target (e.g., S S S). In the incongruent condition, a different letter, randomly selected from among the remaining three, was used as a flanker (e.g., C S C). In the audiovisual condition, the central letter was presented visually, and the flankers were delivered auditorily through headphones. These auditory flankers corresponded to the pronunciation of the target letter in the audiovisual congruent condition and to the pronunciation of a different randomly selected letter in the audiovisual incongruent condition. All letters were presented in black Arial font (25-point size) against a gray background. Each stimulus display lasted 500 ms and was followed by a black screen lasting 2000 ms, resulting in a total trial duration of 2500 ms.

### 2.3. Design and Procedure

#### 2.3.1. Design

**Figure 2:**
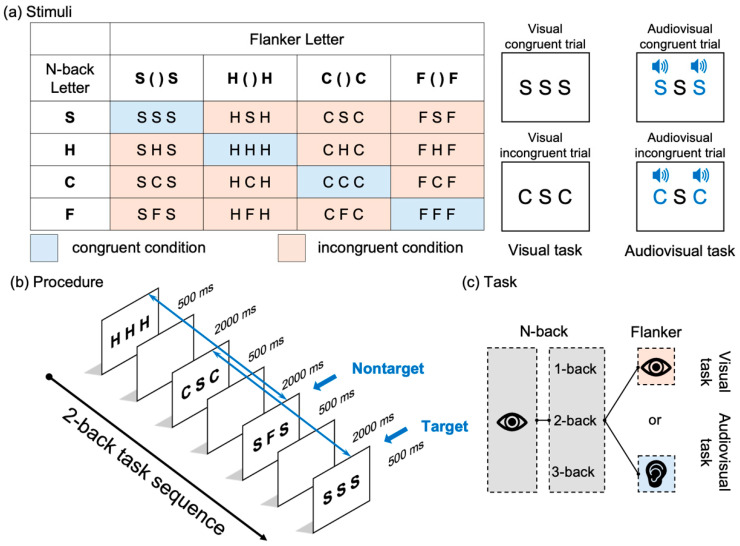
(**a**) Examples of stimulus configurations for the flanker task under visual and audiovisual modalities. Each trial consisted of a centrally presented target letter (S, H, C, or F) and two flanker letters, which were either congruent (same as the target) or incongruent (different from the target). In the visual condition, all letters were presented visually (e.g., S S S or C S C). In the audiovisual condition, the target letter was presented visually while flanker letters were presented auditorily via headphones, corresponding either to the same (congruent) or different (incongruent) letter sounds. (**b**) Trial sequence of the 2-back task. Participants viewed a continuous stream of letter stimuli and responded whether the current target matched the letter presented n trials earlier. Each stimulus appeared for 500 ms, followed by a 2000 ms interstimulus interval. (**c**) Task structure. Participants completed a 2 (modality: visual vs. audiovisual) × 3 (load: 1-back, 2-back, 3-back) within-subjects design.

This study examined how increased working memory load modulates the interaction between updating and inhibition functions under visual (within-modality) and audiovisual (cross-modality) conditions (Figure 2). To assess cognitive processing, both behavioral performance and event-related potentials (ERPs) were recorded. Working memory load was manipulated using a visual n-back task at three levels of difficulty (1-back, 2-back, and 3-back). Inhibitory demands were manipulated through a flanker task that included two interference conditions (congruent and incongruent). The experiment employed two modality conditions. In the visual within-modality condition, visual flanker stimuli were embedded within a visual n-back task. In the audiovisual cross-modality condition, auditory flanker stimuli were embedded within the same visual n-back task (Figure 2a). Thus, the flanker modality (visual vs. auditory) served as a between-condition manipulation of the sensory modality. The overall design had a 2 (modality: visual vs. audiovisual) × 3 (load: 1-back, 2-back, 3-back) within-subjects factorial structure (Figure 2c). The six combinations of modality and WM load were presented in six separate blocks. Each block consisted of a sequence of n-back trials with modality-specific flanker stimuli.

#### 2.3.2. Procedure

The participants performed a visual n-back task in which a temporal sequence of letter stimuli was presented (Figure 2b). During each trial, the participants were instructed to demonstrate whether the centrally presented target letter matched the letter that appeared n steps earlier in the sequence. A match was classified as an n-back target, and a mismatch was classified as an n-back nontarget. Responses were made via a standard QWERTY keyboard: participants pressed the “F” key with their left index finger to indicate a target (match) and the “J” key with their right index finger to indicate a nontarget (mismatch). They were instructed to respond as quickly and accurately as possible, focusing solely on the centrally presented letter while ignoring the flanker stimuli, whether visual or auditory. The stimuli were presented using E-Prime software (E-Prime 3 Professional, Psychology Software Tools, Inc., Sharpsburg, PA, USA), which uses predefined pseudorandomized lists of stimuli. To control for potential conflict adaptation effects—commonly known as the Gratton effect [54,55,56]—sequences that included consecutive incongruent trials (i.e., incongruent followed by incongruent) were excluded during list construction. Additionally, congruent trials following incongruent trials were excluded from the behavioral and ERP analyses [27]. Each block consisted of 150 trials preceded by n null trials. The first n trials of each block were excluded from analysis because no target comparison could be made prior to the nth trial. Excluding these null trials, each block lasted approximately 6.25 min. The order of the experimental blocks was randomized across participants, with short breaks provided between blocks. Half of the trials in each block were targets, and half were nontargets. Within each response category, approximately one-third of the trials were incongruent (e.g., S C S), and two-thirds were congruent (e.g., S S S). Before each experimental block began, the participant completed a short practice session consisting of 15 trials preceded by n null trials. The training was repeated until the participant achieved at least 60% AC.

### 2.4. Data Collection and Analysis

#### 2.4.1. Data Collection

The experiment was conducted in a quiet, dimly lit room. The participants were seated approximately 60 cm from a 22-inch Dell LCD monitor with a screen resolution of 1680 × 1050 pixels. AC and RTs were recorded using E-Prime software (E-Prime 3 Professional, Psychology Software Tools, Inc.). Electroencephalographic (EEG) data were recorded from 32 active electrodes positioned according to the international 10–20 system (Jasper, 1958) using an ActiCHamp amplifier system (Brain Products GmbH, Gilching, Germany). The reference electrode was placed on the right mastoid, and the ground electrode was placed at Fpz. Two additional electrodes were used to record electrooculogram (EOG) activity. One of these was placed below the left eye and used to monitor vertical eye movements (VEOGs), and the other was placed near the outer canthus of the right eye (lateral to the eye) and used to monitor horizontal eye movements (HEOGs). These EOG signals were used to detect and correct for ocular artifacts. EEG signals were acquired using PyCorder software (version 1.0.8) at a sampling rate of 1000 Hz. Electrode impedance was maintained below 5 kΩ throughout the recording. All recordings were made using active electrodes (ActiCap, Brain Products GmbH).

#### 2.4.2. Data Analysis

AC under each task condition was calculated as the mean percentage of correct responses across all trials. Reaction times (RTs) were calculated as the mean RTs for correct responses within each condition. Trials in which the RTs was shorter than 200 ms (<0.05% of all trials) or in which it exceeded ±3 standard deviations of a participant’s condition-specific mean were excluded from RT analysis through the use of SPSS Statistics (version 29.0.1.0; IBM Corp., Armonk, NY, USA). EEG data were preprocessed and analyzed using BrainAnalyzer 2.0 software (Brain Products GmbH, Gilching, Germany). The continuous EEG signal was bandpass-filtered from 0.5 to 40 Hz using a linear finite impulse response (FIR) filter. The epochs were subsequently segmented from −200 ms to 800 ms relative to the onset of the target stimulus, including both congruent and incongruent targets, at each working memory load level. Nontarget trials were excluded from the analysis, and to minimize potential carryover effects, trials in which a congruent target was immediately preceded by an incongruent stimulus were also excluded. Baseline correction was applied using the 200 ms prestimulus interval. Automatic artifact rejection was performed by excluding epochs exceeding ±100 μV in amplitude, and EEG epochs containing eye blinks or minor artifacts were further removed through trial-by-trial visual inspection [57,58]. The P300 component was defined as the maximum positive amplitude within the 250–500 ms poststimulus window and was quantified at electrode Fz. The N200 component was defined as the maximum negative amplitude within the 150–300 ms window and was recorded at electrode Cz. Statistical analyses of the peak amplitudes of both ERP components across conditions were performed.

### 2.5. Statistical Analysis

SPSS Statistics (version 29.0.1.0; IBM Corp.) was used to conduct all of the statistical analyses. For each dependent measure, three-way repeated-measures analysis of variance (ANOVA) was performed with the following within-subject factors: n-back level (1-back, 2-back, 3-back), flanker modality (visual vs. auditory), and flanker congruency (congruent vs. incongruent). Separate ANOVAs were conducted for AC, RTs, and ERP measures (P300 and N200 peak amplitudes). In addition, to assess the flanker interference effect (i.e., incongruent minus congruent), interference scores were subjected to two-way repeated-measures ANOVA with the n-back level and flanker modality as within-subject factors. When significant main effects or interactions were observed, follow-up pairwise comparisons were performed using Bonferroni correction to control for multiple comparisons. Degrees of freedom were adjusted using the Greenhouse–Geisser correction where appropriate, and effect sizes were reported as partial eta squared (*η_p_*^2^).

## 3. Results

A series of three-way repeated measures ANOVAs were conducted to examine the effects of WM load (1-back, 2-back, 3-back), modality configuration (within-modality: visual–visual; cross-modality: visual–auditory), and congruency (congruent, incongruent) on behavioral performance (AC, RTs) and electrophysiological responses (N200 and P300 amplitudes). All three factors were treated as within-subject variables. To further explore how WM load and sensory modality influenced the magnitude of the congruency effect, separate two-way repeated measures ANOVAs of the difference scores (incongruent minus congruent) were conducted for each dependent measure, with WM load and modality again treated as within-subject factors.

### 3.1. Behavioral Results

Only RTs measured in correct trials were included in the analysis. Trials in which the RTs or AC values exceeded ±3 standard deviations from each participant’s mean were excluded.

#### 3.1.1. Accuracy

**Figure 3:**
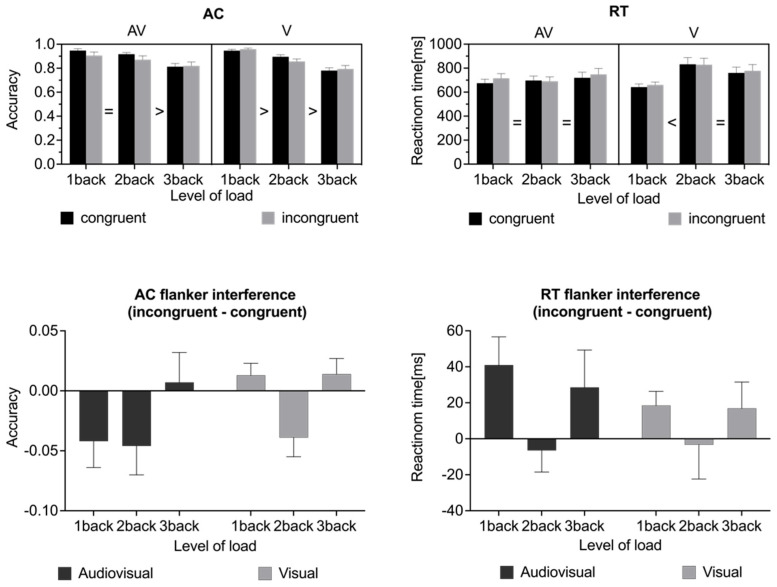
Mean AC and mean RTs for congruent and incongruent flanker trials at three WM load levels (1-back, 2-back, and 3-back) and two modality configurations (audiovisual (AV) and visual (V)). Flanker interference effects are computed as the differences in accuracy and RTs between incongruent and congruent trials (incongruent minus congruent) and are plotted separately for the AV and V conditions.

The AC results (Figure 3) revealed a main effect of the updating load (*F*(2,20) = 15.038, *p* < 0.001, *η_p_*^2^ = 0.532), showing that accuracy decreased as the n-back level increased (1-back: 0.939 ± 0.014, 2-back: 0.885 ± 0.016, 3-back: 0.802 ± 0.025). No significant main effects were found for modality (*F*(1,21) = 0.258, *p* = 0.617, *η_p_*^2^ = 0.012) or congruency (*F*(1,21) = 1.895, *p* = 0.183, *η_p_*^2^ = 0.083). However, there was a significant interaction effect between WM load and modality (*F*(2,20) = 3.710, *p* = 0.033, *η_p_*^2^ = 0.150). Post hoc pairwise comparisons revealed that in the audiovisual condition, AC at 3-back (0.816 ± 0.027) was significantly lower than AC at 1-back (0.926 ± 0.021, *p* = 0.003, 95% CI [−0.183, −0.036]) and 2-back (0.894 ± 0.021, *p* = 0.005, 95% CI [−0.133, −0.022]), with no significant difference between 1-back and 2-back (*p* = 0.255, 95%CI [−0.014, 0.078]). In contrast, AC in the visual condition significantly decreased with increasing n-back level (1-back: 0.953 ± 0.009; 2-back: 0.875 ± 0.018; 3-back: 0.787 ± 0.026, *ps* < 0.05). Additionally, a significant interaction between WM load and congruency was found (*F*(2,20) = 9.443, *p* < 0.001, *η_p_*^2^ = 0.310). Post hoc pairwise comparisons revealed that AC decreased with increasing n-back level under both congruent (1-back: 0.947 ± 0.013, 2-back: 0.906 ± 0.013, 3-back: 0.797 ± 0.024, *ps* < 0.05) and incongruent conditions (1-back: 0.932 ± 0.016, 2-back: 0.863 ± 0.022, 3-back: 0.807 ± 0.027, *ps* < 0.05). Notably, a significant congruency effect was observed only in the 2-back condition (*p* = 0.014, 95% CI [−0.076, −0.009]), in which AC was lower for incongruent trials (0.863 ± 0.022) than for congruent trials (0.906 ± 0.013). No such differences were found in the 1-back (congruent: 0.947 ± 0.013, incongruent: 0.932 ± 0.016, *p* = 0.233, 95% CI [−0.010, 0.040]) or 3-back (congruent: 0.797 ± 0.024, incongruent: 0.807 ± 0.027, *p* = 0.383, 95%CI [−0.035, 0.014]) conditions. No significant interaction between modality and congruency was observed (*F*(1,21) = 0.898, *p* = 0.354, *η_p_*^2^ = 0.041), nor was there a three-way interaction among WM load, modality, and congruency (*F*(2,20) = 3.018, *p* = 0.060, *η_p_*^2^ = 0.126). To further examine flanker interference with AC, a separate two-way repeated-measures ANOVA of the congruency effect (incongruent minus congruent scores) was conducted, with WM load and modality as within-subject factors. Flanker interference with AC was operationalized as the difference in performance between incongruent and congruent trials (i.e., incongruent AC minus congruent AC). A negative value for this parameter indicates that participants made more errors on incongruent trials than on congruent trials, reflecting a disruption of inhibitory control. The larger this negative difference is, the greater is the degree of interference. In contrast, values near zero or positive differences suggest minimal or no flanker interference, potentially reflecting preserved inhibitory control. This analysis revealed a significant main effect of WM load (*F*(2,20) = 9.443, *p* < 0.001, *η_p_*^2^ = 0.310), showing that the congruency effect varied with WM load. The interference effect was more pronounced in the 2-back condition (−0.04 ± 0.02) than in the 1-back (−0.02 ± 0.01, *p* = 0.038, 95% CI [−0.054, −0.002]) and 3-back (0.01 ± 0.01, *p* < 0.001, 95% CI [−0.080, −0.026]) conditions. No significant main effect of modality was found (*F*(1,21) = 0.898, *p* = 0.354, *η_p_*^2^ = 0.041), and no interaction effect between updating load and modality was observed (*F*(2,20) = 3.018, *p* = 0.060, *η_p_*^2^ = 0.126).

#### 3.1.2. Reaction Times

A main effect of updating load (*F*(2,20) = 6.148, *p* = 0.005, *η_p_*^2^ = 0.226) suggested that RTs were significantly shorter in the 1-back condition than in the 2-back condition (*p* = 0.004, 95%CI [−150.96, −27.219]); there was no significant difference between any other two conditions (1-back: 673.05 ± 27.77 ms, 2-back: 762.136 ± 43.13 ms, 3-back: 751.30 ± 46.90 ms). A main effect of modality was observed (*F*(1,21) = 5.937, *p* = 0.024, *η_p_*^2^ = 0.220, 95% CI [−79.20, −6.26]), with significantly shorter RTs for audiovisual flanker trials (707.46 ± 36.31 ms) than for visual flanker trials (750.19 ± 39.29 ms). A main effect of congruency was also found (*F*(1,21) = 6.69, *p* = 0.017, *η_p_*^2^ = 0.242, 95%CI [3.11, 28.60]), indicating that RTs were longer for incongruent trials (736.75 ± 38.05 ms) than for congruent trials (720.90 ± 35.76 ms). Moreover, a significant interaction effect between updating load and modality was found (*F*(2,20) = 10.765, *p* < 0.001, *η_p_*^2^ = 0.339). Post hoc pairwise comparisons with Bonferroni correction revealed no significant differences in RTs across the three load levels under the audio-visual condition (1-back: 694.75 ± 35.85 ms, 2-back: 694.07 ± 35.79 ms, 3-back: 733.57 ± 47.36 ms, *ps* > 0.05). However, under the visual condition, the RTs in the 2-back (830.21 ± 54.25 ms, *p* < 0.001, 95% CI [78.84, 278.90]) and 3-back (769.02 ± 49.83 ms, *p* = 0.022, 95% CI [14.40, 220.96]) conditions were both significantly longer than the RTs in the 1-back (651.34 ± 25.29 ms) condition, with no significant difference between the 2-back and 3-back conditions (*p* = 0.295, 95% CI [−30.84, 153.21]). In terms of the modality effect, RTs were significantly shorter for audiovisual flanker trials (694.07 ± 35.80 ms) than for visual flanker trials (830.21 ± 54.25 ms) only in the 2-back condition (*F*(1,21) = 18.380, *p* < 0.001, *η_p_*^2^ = 0.467, 95% CI [−202.17, −70.10]). No significant modality effects were found in the 1-back (audiovisual: 694.75 ± 35.85 ms, visual: 651.341 ± 25.29 ms, *p* = 0.131, 95% CI [−14.07, 100.89]) or 3-back conditions (audiovisual: 733.57 ± 47.36 ms, visual: 769.02 ± 49.83 ms, *p* = 0.180, 95% CI [−88.67, 17.76]). Finally, the three-way interaction effect among updating load, modality and congruency was not significant (*F*(2,20) = 0.322, *p* = 0.726, *η_p_*^2^ = 0.015). The flanker interference effect on RTs reflects the extent to which task-irrelevant distractors slow behavioral responses. It is typically operationalized as the difference in RTs between incongruent and congruent trials, with larger differences indicating greater interference. A positive interference score implies that incongruent flankers impede performance, whereas a negative or near-zero score may indicate reduced interference or the absence of interference. In contrast to the findings for AC, analysis of the flanker interference effect on RTs revealed no significant main effect of WM load (*F*(2,20) = 2.668, *p* = 0.081, *η_p_*^2^ = 0.113) or modality (*F*(1,21) = 0.767, *p* = 0.391, *η_p_*^2^ = 0.035) and no significant interaction effect between updating load and modality (*F*(2,20) = 0.320, *p* = 0.728, *η_p_*^2^ = 0.015).

### 3.2. ERP Results

**Figure 4:**
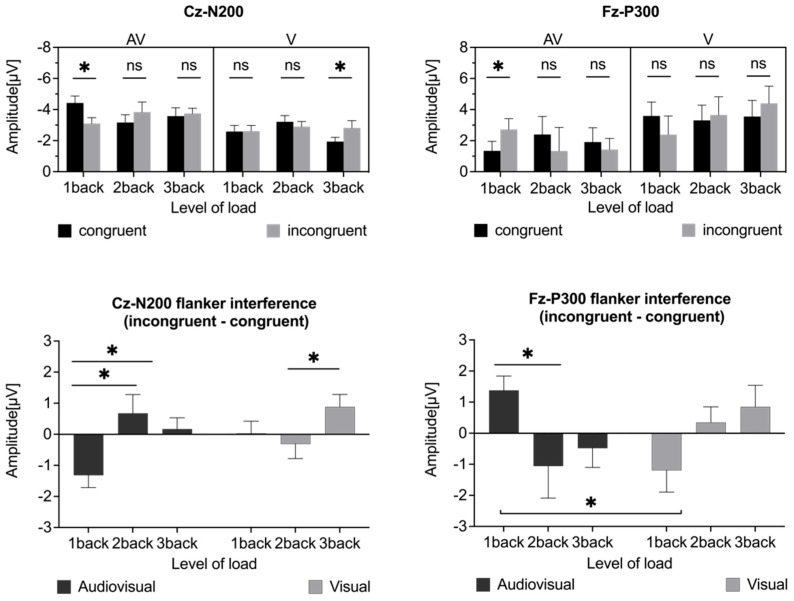
Event-related potential (ERP) amplitudes and flanker interference effects for the N200 and P300 components under varying WM load and modality conditions. The mean amplitudes of the N200 component at electrode Cz (Cz–N200) and the mean amplitudes of the P300 component at electrode Fz (Fz–P300) for congruent (black bars) and incongruent (gray bars) flanker trials across three WM load levels (1-back, 2-back, 3-back) are presented separately for audiovisual (AV) and visual (V) modality conditions. Flanker interference effects were computed as the difference in amplitude between incongruent and congruent trials (incongruent minus congruent) and are shown separately for the AV and V conditions at each load level. *: *p* < 0.05, ns: not significant.

**Figure 5:**
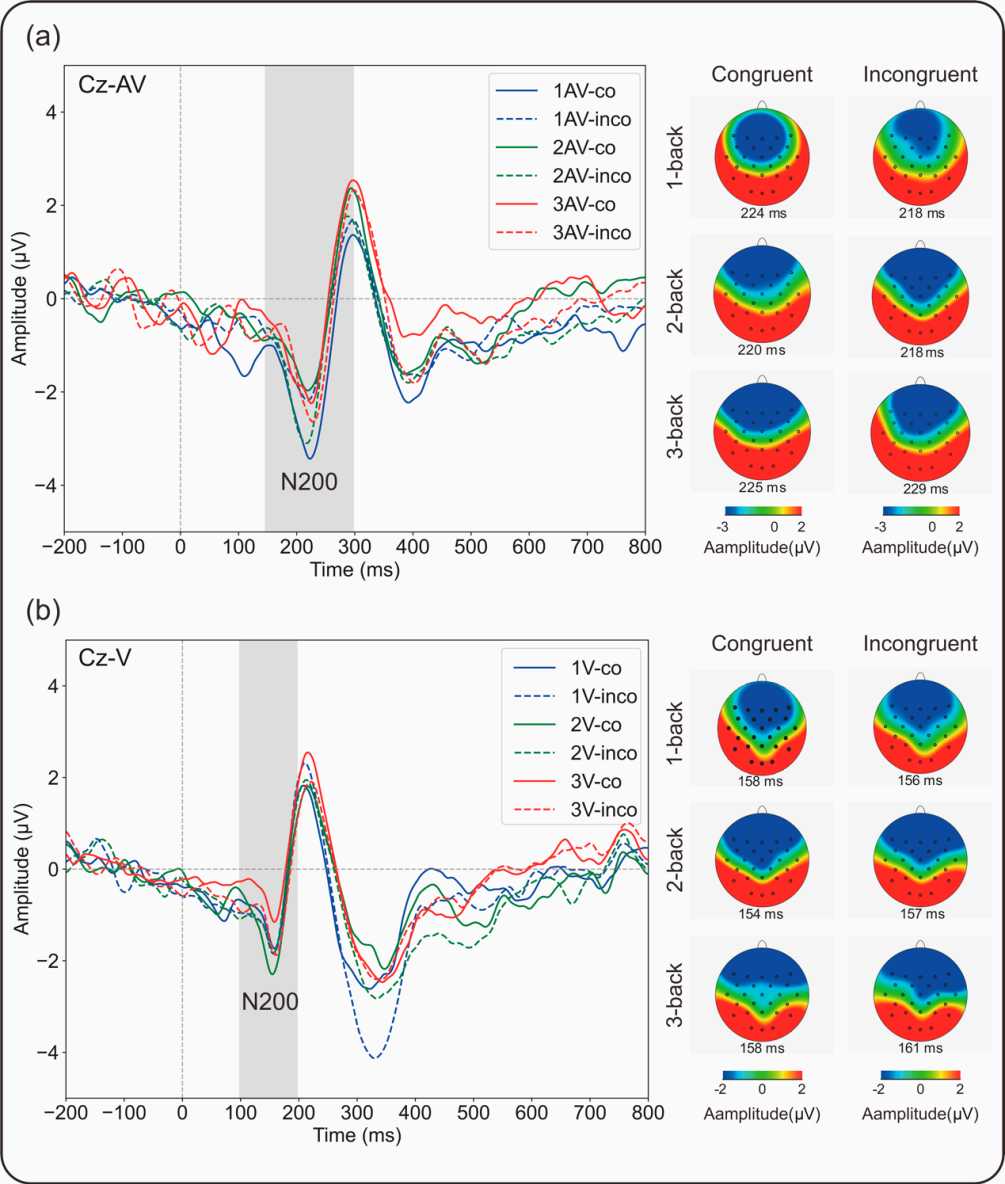
Grand-averaged ERP waveforms at electrode Cz for (**a**) audiovisual (Cz–AV) and (**b**) visual (Cz–V) flanker tasks at three WM load levels (1-back, 2-back, and 3-back) and two flanker congruency conditions (congruent and incongruent). The shaded area indicates the N200 time window. The scalp topographies illustrate the N200 distribution across conditions; the rows show increasing WM load (top to bottom: 1-back, 2-back, and 3-back), and the columns show congruent (**left**) and incongruent (**right**) trials.

**Figure 6:**
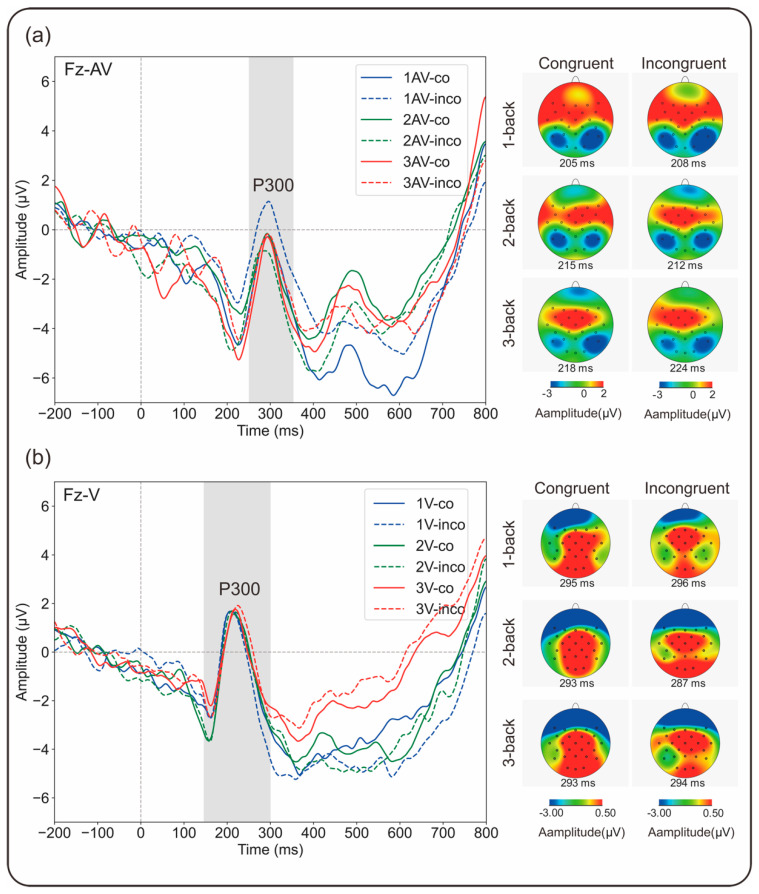
Grand-averaged ERP waveforms at electrode Fz for (**a**) audiovisual (Fz–AV) and (**b**) visual (Fz–V) flanker tasks across three WM load levels (1-back, 2-back, and 3-back) and two flanker congruency conditions (congruent and incongruent). The shaded area indicates the P300 time window. The scalp topographies illustrate the P300 distribution across conditions; the rows represent increasing WM load (top to bottom: 1-back, 2-back, 3-back), and the columns represent congruent (**left**) and incongruent (**right**) trials.

Separate three-way repeated-measures analyses of variance (ANOVAs) of the mean amplitudes of the N200 and P300 components were conducted. The mean values of all the dependent variables are summarized in Figure 4. Grand-average ERP waveforms and corresponding topographic distributions are presented in Figure 5 and Figure 6. To further examine flanker interference at the neural level, we calculated difference scores between incongruent and congruent trials (incongruent minus congruent) for both the N200 and P300 components. For N200, more negative amplitude in the incongruent condition reflects enhanced conflict monitoring, indicative of a stronger flanker interference effect. For P300, reduced amplitude in the incongruent condition implies a greater demand for cognitive control and reduced attentional allocation. In both cases, larger differences reflect stronger interference effects. These results are illustrated in Figure 4, which displays the interference effect magnitudes across the WM load and modality conditions.

#### 3.2.1. N200 Amplitude

The ERP waveforms at electrode Cz are shown in Figure 5, and the mean amplitude values are presented in Figure 4. A significant main effect of modality was observed (*F*(1,21) = 8.585, *p* = 0.008, *η_p_*^2^ = 0.290, 95% CI [−1.645, −0.279]), with a larger N200 amplitude for the audiovisual condition (−3.64 ± 0.35 µV) than for the visual condition (−2.68 ± 0.26 µV). No significant main effects were found for the WM updating load (*F*(2,20) = 0.581, *p* = 0.564, *η_p_*^2^ = 0.027) or for congruency (*F*(1,21) = 0.017, *p* = 0.896, *η_p_*^2^ = 0.001). However, there was a significant interaction effect between the updating load and congruency (*F*(2,20) = 3.377, *p* = 0.044, *η_p_*^2^ = 0.139). Post hoc pairwise comparisons with Bonferroni correction indicated a smaller N200 amplitude for incongruent (−2.86 ± 0.26 µV) than for congruent (−3.50 ± 0.34 µV) flanker trials in the 1-back condition (*p* = 0.035, 95% CI [0.048, 1.241]), a larger N200 amplitude for incongruent (−3.28 ± 0.33 µV) than for congruent (−2.75 ± 0.31 µV) flanker trials in the 3-back condition (*p* = 0.022, 95% CI [−0.966, −0.085]), and no significant difference in the 2-back condition (congruent: −3.18 ± 0.29 µV, incongruent: −3.37 ± 0.45 µV, *p* = 0.656, 95% CI [−0.658, 1.022]). When N200 amplitudes at various levels of updating load were compared, no significant differences were observed for either congruent (1-back: −3.50 ± 0.34 µV, 2-back: −3.18 ± 0.29 µV, 3-back: −2.75 ± 0.31 µV, *ps* > 0.05) or incongruent flanker trials (1-back: −2.86 ± 0.26 µV, 2-back: −3.37 ± 0.45 µV, 3-back: −3.28 ± 0.33 µV, *ps* > 0.05). A significant three-way interaction among updating load, modality, and congruency was observed (*F*(2,20) = 3.473, *p* = 0.040, *η_p_*^2^ = 0.142). Post hoc pairwise comparisons with Bonferroni correction indicated that in the audiovisual task, incongruent flanker trials (−3.10 ± 0.38 µV) elicited significantly smaller N200 amplitudes than did congruent flanker trials (−4.42 ± 0.44 µV) in the 1-back condition (*p* = 0.003, 95% CI [0.492, 2.143]), with no significant differences in the 2-back condition (congruent: −3.16 ± 0.50 µV, incongruent: −3.83 ± 0.65 µV, *p* = 0.275, 95% CI [−0.577, 1.926]) or in the 3-back condition (congruent: −3.57 ± 0.53 µV, incongruent: −3.74 ± 0.35 µV, *p* = 0.642, 95% CI [−0.581, 0.921]). In the visual task, incongruent flanker trials (−2.82 ± 0.47 µV) elicited significantly larger N200 amplitudes than did congruent flanker trials (−1.94 ± 0.28 µV) in the 3-back condition (*p* = 0.039, 95% CI [−1.713, −0.048]), with no significant differences in the 1-back (congruent: −2.58 ± 0.28 µV, incongruent: −2.61 ± 0.47 µV, *p* = 0.944, 95% CI [−0.789, 0.845]) or 2-back conditions (congruent: −3.21 ± 0.41 µV, incongruent: −2.90 ± 0.33 µV, *p* = 0.516, 95% CI [−1.287, 0.667]). The flanker interference effect on the mean amplitude of N200 is defined as the difference in mean N200 amplitude between incongruent and congruent trials (incongruent minus congruent). A larger (more negative) amplitude in the incongruent condition than in the congruent condition indicates the presence of a flanker effect. In this context, greater differences in the values reflect stronger interference. Conversely, a smaller N200 amplitude in incongruent trials than in congruent trials suggests the absence of a typical flanker interference effect. A significant main effect of updating load was observed (*F*(2,20) = 3.371, *p* = 0.044, *η_p_*^2^ = 0.138), indicating greater flanker interference for the 3-back condition than for the 1-back condition (*p* = 0.009, 95% CI [−2.012, −0.326]), with no significant difference between any other two conditions (1-back: 0.64 ± 0.28 µV, 2-back: −0.18 ± 0.40 µV, 3-back: −0.53 ± 0.21 µV). The main effect of modality was not significant (*F*(1,21) = 1.021, *p* = 0.324, *η_p_*^2^ = 0.046, 95% CI [−0.378, 1.092]). However, the interaction effect between updating load and modality was significant (*F*(2,20) = 3.478, *p* = 0.040, *η_p_*^2^ = 0.142). Post hoc pairwise comparisons with Bonferroni correction revealed that in the 1-back condition (*p* = 0.022, 95% CI [0.217, 2.476]), the flanker interference effect was significantly smaller for the audiovisual task (1.32 ± 0.40 µV) than for the visual task (–0.03 ± 0.39 µV), with no significant differences in the 2-back (*p* = 0.183, 95% CI [−2.475, 0.504]) or 3-back (*p* = 0.275, 95% CI [−0.609, 2.029]) conditions. In terms of the updating load effect, under the audiovisual condition, there was greater flanker interference for the 2-back (−0.68 ± 0.60 µV, *p* = 0.018, 95% CI [−3.609, −0.376]) and 3-back conditions (−0.17 ± 0.36 µV, *p* < 0.001, 95% CI [−2.221, −0.753]) than for the 1-back condition (1.32 ± 0.40 µV), with no significant difference between the 2-back and 3-back conditions (*p* = 0.514, 95% CI [−2.090, 1.079]). In contrast, under the visual condition, there was a significant difference only between the 2-back and 3-back conditions(*p* = 0.046, 95% CI [0.026, 2.355]), indicating that the flanker effect for the 3-back condition was significantly greater than that for the 2-back condition (1-back: −0.03 ± 0.40 µV, 2-back: 0.31 ± 0.47 µV, 3-back: −0.88 ± 0.40 µV) and revealing a sharper increase in interference under higher loads.

#### 3.2.2. P300 Amplitude

The ERP curves obtained at electrode Fz are shown in Figure 6, and the mean amplitude values at this electrode are given in Figure 4. No significant main effects of updating load (*F*(2,20) = 0.261, *p* = 0.772, *η_p_*^2^ = 0.012) or congruency (*F*(1,21) = 0.015, *p* = 0.905, *η_p_*^2^ = 0.001) were found. However, a significant main effect of modality was observed (*F*(1,21) = 8.302, *p* = 0.009, *η_p_*^2^ = 0.283, 95% CI [−2.799, −0.452]), indicating a smaller P300 amplitude for audiovisual flanker trials (1.86 ± 0.79 µV) than for visual flanker trials (3.48 ± 0.95 µV). There were no significant two-way interactions, but a significant three-way interaction among updating load, modality, and congruency was observed (*F*(2,20) = 3.833, *p* = 0.030, *η_p_*^2^ = 0.154). Post hoc pairwise comparisons with Bonferroni correction suggested that, in congruent flanker trials, audiovisual flanker trials (1.34 ± 0.62 µV) elicited significantly smaller P300 amplitudes than did visual flanker trials (3.59 ± 0.89 µV) in the 1-back condition (*p* = 0.005, 95% CI [−3.733, −0.762]). No significant difference in P300 amplitude was found between the 2-back (audiovisual: 2.40 ± 1.16 µV, visual: 3.31 ± 0.98 µV, *p* = 0.434, 95% CI [−3.285, 1.463]) and 3-back conditions (audiovisual: 1.91 ± 0.91 µV, visual: 3.55 ± 1.04 µV, *p* = 0.056, 95% CI [−3.322, 0.048]). In incongruent flanker trials, the P300 amplitude for audiovisual flanker trials (1.43 ± 0.72 µV) was significantly smaller than that for visual flanker trials (4.40 ± 1.11 µV) in the 3-back condition (*p* = 0.019, 95% CI [−5.402, −0.534]). No significant difference was found between the 1-back (audiovisual: 2.72 ± 0.70 µV, visual: 2.39 ± 1.20 µV, *p* = 0.765, 95% CI [−1.918, 2.572]) and 2-back conditions (audiovisual: 1.34 ± 1.52 µV, visual: 3.65 ± 1.17 µV, *p* = 0.055, 95% CI [−4.683, 0.052]). In terms of the congruency effect, in the audiovisual flanker trials, incongruent flanker trials (2.72 ± 0.70 µV) elicited significantly larger P300 amplitudes than did congruent flanker trials (1.34 ± 0.62 µV) in the 1-back condition (*p* = 0.007, 95% CI [0.425, 2.333]), with no significant difference in the 2-back condition (congruent: 2.40 ± 1.16 µV, incongruent: 1.34 ± 1.52 µV, *p* = 0.315, 95% CI [−1.082, 3.199]) or the 3-back condition (congruent: 1.91 ± 0.91 µV, incongruent: 1.43 ± 0.72 µV, *p* = 0.444, 95% CI [−0.805, 1.771]). In visual flanker trials, the P300 amplitude was smaller in incongruent flanker trials (2.40 ± 1.20 µV) than in congruent flanker trials (3.59 ± 0.89 µV) in the 1-back condition (*p* = 0.103, 95% CI [−2.652, 0.262]) but larger in the 2-back (congruent: 3.31 ± 0.98 µV, incongruent: 3.65 ± 1.17 µV, *p* = 0.497, 95% CI [−0.696, 1.387]) and 3-back conditions (congruent: 3.55 ± 1.04 µV, incongruent: 4.40 ± 1.11 µV, *p* = 0.235, 95% CI [−0.593, 2.289]), with no significant difference between the P300 amplitudes for congruent and incongruent flanker trials at any level of updating. The flanker interference effect on the mean amplitude of P300 is defined as the difference in mean P300 amplitude between incongruent and congruent trials (incongruent minus congruent). A smaller (less positive) amplitude in the incongruent condition than in the congruent condition indicates the presence of a flanker effect. In this context, greater differences reflect stronger interference. Conversely, a larger P300 amplitude in incongruent trials than in congruent trials implies the absence of a typical flanker interference effect. No significant main effects of updating load (*F*(2,20) = 0.370, *p* = 0.693, *η_p_*^2^ = 0.017) or modality (*F*(1,21) = 0.014, *p* = 0.908, *η_p_*^2^ = 0.001) were found. However, there was a significant interaction effect between updating load and modality (*F*(2,20) = 3.833, *p* = 0.030, *η_p_*^2^ = 0.154). Post hoc pairwise comparisons with Bonferroni correction indicated that the flanker interference effect for the audiovisual task (1.38 ± 0.46 µV) was significantly smaller than that for the visual task (−1.20 ± 0.70 µV) in the 1-back condition (*p* = 0.021, 95% CI [0.422, 4.727]), with no significant difference in the 2-back (*p* = 0.209, 95% CI [−3.658, 0.849]) or 3-back (*p* = 0.217, 95% CI [−3.505, 0.843]) conditions. In terms of updating loads, in the audiovisual task, the flanker interference effect was significantly smaller in the 1-back condition than in the 3-back condition (*p* = 0.037, 95% CI [0.128, 3.596]), with no significant difference between any other two conditions (1-back: 1.38 ± 0.46 µV, 2-back: −1.06 ± 1.03 µV, 3-back: −0.48 ± 0.62 µV). In the visual task, no significant difference in flanker interference was found across load levels (1-back: −1.20 ± 0.70 µV, 2-back: 0.35 ± 0.50 µV, 3-back: 0.85 ± 0.70 µV, *ps* > 0.05).

## 4. Discussion

The present study extends prior investigations of the interplay between concurrent updating and inhibitory control by examining whether such interactions are sensory modality-specific. We investigated how updating load, manipulated via a visual verbal n-back task, modulates inhibitory control demands elicited by visual (within-modality) or auditory (cross-modality) verbal flanker stimuli under varying WM loads. Our findings demonstrate that sensory input modality modulates both behavioral performance and neural dynamics in the interaction between concurrent WM functions, as evidenced by load-sensitive behavioral and ERP indices of updating and inhibition.

The behavioral results (Figure 3) revealed that greater WM load (2- and 3-back) impaired task performance, as indicated by significantly lower AC and longer RTs in the 2-back and 3-back tasks than in the 1-back task across both the within-modality and cross-modality conditions. While no significant difference was found between the two modality conditions under the 1-back load, a divergence between the two conditions began to emerge at the 2-back level. Specifically, the largest flanker interference effect in the 2-back condition led to significantly reduced AC and increased RTs in the visual within-modality task, whereas such effects did not emerge in the audiovisual cross-modality task until the WM load increased to 3-back. Compared with the within-modality condition, significant performance impairment in the cross-modality condition appeared at higher WM load. Whether this difference reflects sensory modality-specific interaction patterns needs to be further examined at the neural level.

ERP results revealed modulations in the N200 and P300 components, which are commonly associated with conflict detection and attentional resource allocation. These modulations may reflect the underlying neural dynamics of updating and inhibition processes that are differentially engaged depending on the modality configuration and the WM load [38,39]. The N200 component (Figure 4), which is commonly referred to as the conflict N200 in the flanker task, typically has a greater (i.e., more negative) amplitude in incongruent trials than in congruent trials [42,44,45,46,59]. The presence of the conflict N200 component in the flanker task has been interpreted as evidence for inhibition of the distracting flankers, which then enables execution of the correct response [46]. However, in the audiovisual cross-modality condition, the expected increase in N200 amplitude for incongruent compared with congruent flankers was not observed. Instead, at a low WM load (1-back), incongruent flankers elicited a significantly smaller (i.e., less negative) N200 amplitude, reflecting a reversed pattern relative to the expected conflict-related effect that is considered to reflect reduced flanker interference. At higher WM loads (2- and 3-back), no significant differences in N200 amplitude between incongruent and congruent flankers were found. In contrast, a significant enhancement in the N200 amplitude was observed at 3-back in the visual within-modality condition. Taken together, the results reveal that increasing the WM load within the same sensory modality leads to stronger flanker interference. In the cross-modality condition, however, no corresponding increase in the interference effect was found as the WM load increased. This observation implies that increasing WM demands elicit sensory modality-specific neural dynamics during the processing of conflicting information. In addition, under low WM load (1-back), a reduced flanker interference effect was observed in the cross-modality condition, as indicated by attenuation of the N200 amplitude for incongruent rather than congruent flankers. This finding might lead to the conclusion that “control N200’’ is frequently mixed with an N200 that is driven by perceptual mismatch [46]. In other words, cross-modality incongruent flankers may elicit the N200 component due to conflict perceptual detection or conflict inhibition control processes. Specifically, under a low WM load (1-back), although cross-modality incongruent information is perceived, the ample cognitive resources available may render inhibition of the conflict less demanding. In such cases, the presence of modality-based conflict may enhance the salience of content-related interference, allowing the cognitive system to classify and disregard incongruent distractors quickly as irrelevant without engaging the resource-intensive inhibitory control processes that are typically required under higher cognitive demand. Conversely, congruent flankers lacking distinctive features for distractor identification may be less readily identified, leading to increased N200 amplitudes owing to the heightened need for conflict monitoring despite the apparent congruency. These N200-related findings offer evidence that the interaction between concurrent executive functions—such as updating and inhibition—is modulated both by bottom-up stimulus-driven perception processes and by top-down cognitive control mechanisms, as demonstrated in prior research [49]. Notably, this modulation appears to differ depending on the modality configuration. While N200 primarily reflects early-stage, perceptually driven conflict detection and inhibition, further examination of the P300 component is needed to understand how top-down attentional selection and inhibitory control are recruited under different WM loads and modality configurations.

P300 serves as an index of the internal distribution of attention when different executive WM functions are required [43]. Its patterns of modulation may reveal how cognitive control processes adapt to varied WM loads across modality-specific contexts. Consistent with the results obtained for the N200 component, in the cross-modality condition (Figure 4 and Figure 5), a reduced flanker interference effect was observed at 1-back, as indicated by a greater P300 amplitude in the presence of incongruent than in the presence of congruent flankers. This result is consistent with the view of the P300 amplitude as a marker of mental workload and reflects the greater amount of attentional resources allocated to interference trials [51,52,53,60,61]. In contrast to studies of visual within-modality processing, in which a smaller P300 amplitude was reported for incongruent than for congruent flankers, when different EFs are necessary for task performance, attention is thought to be distributed among updating and inhibition functions, and presentation of more challenging incongruent flankers decreases the cognitive resources available for target attention and increases the cognitive resources available for interference inhibition [27,47]. Although not statistically significant, a numerical tendency toward a smaller P300 amplitude for incongruent flankers at 1-back was observed in the visual within-modality condition. This finding might support modality-specific modulation patterns of P300 at low WM load levels. The modulation patterns at higher WM load levels also varied systematically between the visual within-modality and audiovisual cross-modality conditions. Notably, a striking reversal in the relative P300 amplitudes between congruent and incongruent flankers emerged in the 2-back condition and remained consistent in the 3-back condition. This finding suggested that, regardless of WM load, the attentional resource allocation patterns during interaction processing of updating and inhibition functions differed. In addition, a significantly smaller P300 amplitude for visual within-modality incongruent flankers than for audiovisual cross-modality incongruent flankers was observed in the 1-back condition. As previously noted for the N200 results, audiovisual cross-modality incongruent flankers under low WM load elicited stronger neural responses, a pattern that appears to extend to later-stage processing, as reflected in the P300 amplitude. These findings demonstrate that at low WM load, inhibition associated with auditory–verbal flankers results in the recruitment of more attentional resources than does inhibition associated with visual–verbal flankers. Furthermore, the present study revealed that increasing the WM load led to increased P300 amplitude in the within-modality condition and decreased P300 amplitude in the cross-modality condition for incongruent flankers. This opposite pattern reveals that, during the interaction between updating and inhibition functions, the strategies for attentional resource allocation differ across sensory modality configurations in response to increasing cognitive demands. At a low WM load, inhibition of auditory–verbal flankers recruits more attentional resources than does inhibition of visual–verbal flankers. As WM load increases, specifically in the cross-modality condition, visual–verbal updating begins to dominate in the allocation of attentional resources, and there is a reallocation of attentional resources from auditory–verbal inhibition (the secondary task) to visual–verbal updating (the primary task) [43,53,62,63,64,65]. Elevated visual–verbal WM load may facilitate the suppression of auditory distractors by reducing selective attentional engagement with auditory input, as evidenced by the decrease in the amplitude of P300 [66] and in auditory brainstem responses (ABRs) [67] to irrelevant auditory stimuli as the load of a visual–verbal WM updating task increases. This pattern is consistent with previous findings that suggest that shielding WM from distraction generally requires more effort than does flexibly updating WM content [68]. Conversely, in the within-modality condition, increasing the WM load appears to recruit additional attentional resources that enhance inhibitory control [27,69], as reflected in the increased P300 amplitude.

Overall, the present findings imply that the interaction between updating and inhibition functions in WM is not uniform but rather is systematically modulated by both cognitive load and sensory modality. As WM demands increase, these executive processes engage sensory-specific interaction mechanisms, resulting in distinct coordination patterns under within-modality and cross-modality conditions. These results point to the existence of modality-specific mechanisms that modulate the allocation of attentional and cognitive resources when multiple executive functions are concurrently engaged and emphasize the importance of incorporating sensory modality into models of executive function interaction. Furthermore, since the current study focused exclusively on verbal information processed through visual and auditory modalities, it remains an open question whether similar patterns would be detected under conditions involving spatial information or other types of representational content processed across different sensory modality pairings. In addition, recent advances in computational and connectivity-based EEG analyses have provided important insights into how distributed neural systems coordinate executive control. For instance, Al-Ezzi et al. (2024) demonstrated that EEG-based effective connectivity, particularly directed information flow between frontal and temporal regions, is sensitive to executive operations such as task switching [70]. These findings suggest that increased frontal connectivity may reflect compensatory recruitment mechanisms that support executive functioning under increased cognitive demand. Integrating such approaches with the present ERP findings could extend our understanding beyond localized temporal dynamics (e.g., N200 and P300) to reveal the directional coordination and network communication underlying executive control across sensory modalities. Addressing these questions will be essential in refining our understanding of how the cognitive system flexibly manages multiple executive demands in complex environments.

## 5. Limitations and Future Directions

The current study had several limitations that need to be considered and addressed in future research. First, the use of a restricted stimulus set (four letters) may have led to potential learning or familiarity effects; future studies could address this by expanding the range of stimuli. Second, as the analyses were limited to the sensor level, future work could incorporate source-localization techniques to obtain more spatially precise insights into the cortical networks supporting the sensory modality-dependent interplay between updating and inhibition. In addition, future research should extend this line of work by conducting large-sample cognitive training focusing on the interplay between updating and inhibition. Building on the current task, such training approaches may help clarify how cognitive training shapes the plasticity of executive control functions.

## Data Availability

The data that support the findings of this study are available from the authors upon reasonable request. The data are not publicly available due to institutional policy restrictions. However, the corresponding author can provide the data upon reasonable request, in accordance with applicable restrictions.
